# Validation of the Hungarian version of the General Oral Health Assessment Index (GOHAI) in clinical and general populations

**DOI:** 10.1186/s12903-024-05198-2

**Published:** 2024-11-19

**Authors:** Judit Oszlánszky, Károly Mensch, Péter Hermann, Zsombor Zrubka

**Affiliations:** 1 Department of Prosthodontics, Faculty of Dentistry, Semmelweis University, Szentkirályi street 47, Budapest, Hungary; 2 Department of Oral Diagnostics, Faculty of Dentistry, Semmelweis University, Szentkirályi street 47, Budapest, Hungary; 3 Health Economics Research Center, University Research and Innovation Center, University of Óbuda, Budapest, Hungary

**Keywords:** Oral health, Quality of life, GOHAI, Psychometrics, Patient-reported outcomes, dPRO, Validation, COSMIN

## Abstract

**Background:**

COSMIN (Consensus-based Standards for the selection of health Measurement INstruments) provides a framework for selecting and validating patient-reported outcome measurements (PROMs). This study aims to validate the Hungarian version of the GOHAI and, for the first time, to assess its Standard Error of Measurement (SEM), Smallest Detectable Change (SDC), and Measurement Invariance (MI) across general and clinical populations as well as different age groups, following COSMIN guidelines.

**Materials and methods:**

The translation was performed using a forward-backward process. A mixed sample (*n* = 306) was recruited in Budapest from May 2023 to February 2024, consisting of the general population (45.1%), recruited from health kiosks and a nursing home, and the clinical population (54.9%), sourced from Semmelweis University’s care units. The sample was further divided into two age groups: 18–64 years old (54.9%) and 65 + years old (45.1%). GOHAI was administered twice to 108 stable participants. For both the additive score (ADD-GOHAI) and simple count (SC-GOHAI), structural validity and measurement invariance by subgroups were assessed via Confirmatory Factor Analysis (CFA). Internal consistency was evaluated using Cronbach’s alpha, and test-retest reliability was measured using the intraclass correlation coefficient (ICC). SEM was calculated using the SEM agreement formula, and SDC using: $\:SDC=1.96*\sqrt{2}*SEM$. Convergent and known-group validity were tested against predefined hypotheses for structural validity.

**Results:**

Contrary to a three factor model, a single-factor model showed good fit in all subgroups for both scoring methods, with adequate internal consistency (Cronbach 𝛼: 0.76–0.85). Four of the six hypotheses for convergent validity and all ten hypotheses for known-groups validity supported the predefined criteria. Measurement invariance between clinical and general populations, or by age, was not demonstrated, so GOHAI’s different measurement properties should be considered when comparing subpopulations. Test-retest reliability was adequate (ICC: 0.87–0.96). SDC was ≈5 points using ADD-GOHAI and 2–3 points using SC-GOHAI.

**Conclusion:**

The Hungarian version of GOHAI demonstrates satisfactory psychometric properties across both general and clinical populations, as well as among both younger and older age groups. While the measurement properties of SC-GOHAI may be more stable between populations, ADD-GOHAI seems more suitable for individual follow-up. However, observed changes must be considered in relation to the measurement error associated with GOHAI.

**Supplementary Information:**

The online version contains supplementary material available at 10.1186/s12903-024-05198-2.

## Introduction

In dental practice, researchers often explore how various treatments affect patient quality of life [1, 2]. With the rise of digital workflows, assessing the impact of modern procedures on dental patient-reported outcomes (dPROs) is increasingly relevant.

A key dPRO is Oral Health-Related Quality of Life (OHRQoL), which includes orofacial function, pain, appearance, and psychosocial impact. The most commonly used OHRQoL measures are the 14-item version of the Oral Health Impact Profile (OHIP) [3, 4] and the General Oral Health Assessment Index (GOHAI) [5, 6]. Developed in the 1990s, these tools have been validated in multiple languages [7]. Comparative studies show that both instruments have good psychometric properties. While both measure OHRQoL, they differ in focus: OHIP emphasizes the psychosocial domain, while GOHAI focuses on functionality and dental status [8–11].

Over the last decades, the methodological standards of psychometric research have evolved significantly, accompanied by changes in the taxonomy of measurement properties of patient-reported outcome measures (PROMs). The COSMIN (Consensus-based Standards for the selection of health Measurement INstruments) provides a framework for selecting PROMs and guiding their validation process [12–14]. To date, only one COSMIN systematic review has summarized the psychometric properties associated with GOHAI and highlighted several deficiencies [15]. In addition to methodological shortcomings, certain psychometric properties, such as responsiveness and cross-cultural validity, remain largely unexplored. However, neither GOHAI nor OHIP has undergone a validation study following the COSMIN protocol.

Cross-cultural adaptation is crucial because many cultural factors influence oral health perception. Differences in culture, care accessibility, and national characteristics make thorough cultural adaptation necessary, extending beyond mere translation [16, 17].

Reliable research depends on accurate measurement tools. Validated PROMs are crucial for evaluating healthcare interventions, understanding patient perspectives, and guiding clinical and policy decisions. While several measurement properties of GOHAI have extensively been evaluated, this is the first study to examine the standard error of measurement (SEM), smallest detectable change (SDC), and measurement invariance (MI).

According to COSMIN and classical test theory (CTT), the SEM based on a test-retest design is the preferred statistic for measuring measurement error. Changes below the SDC indicate measurement error, and those above suggest real changes. MI examines if different groups with the same trait level respond similarly. It is essential to compare populations and interpret results accurately. Dental care is primarily provided by private practices in Hungary, and dental problems of various severity are ubiquitous among the general population [18]. Furthermore, the use of GOHAI has been gradually extended from geriatric patients to the general population [19]. Hence, it is important to explore whether GOHAI measures OHRQoL similarly across these groups.

This study aims, for the first time in the literature, to validate the Hungarian version of the GOHAI according to COSMIN guidelines and to assess its SEM, SDC, and measurement invariance between the general and clinical populations as well as across different age groups.

## Methods

### The general oral health assessment index

The questionnaire consists of 12 items assessing three hypothesized dimensions: ‘physical function’, ‘psycho-social function’, and ‘pain and discomfort’. The questions of GOHAI focus on the last 3 months, and its items are scored on either 3, 5, or 6-level Likert-type frequency scales. Two different scores of the GOHAI can be calculated. The additive score (ADD-GOHAI) is the sum of Likert scores after reversing the oppositely worded items. Its range is 12–36, 12–60, and 0–60 when using the 3, 5, and 6-level Likert items, respectively (high scores indicate few problems). The simple count score (SC-GOHAI) counts items with the responses ‘sometimes,’ ‘often’, and ‘always’ and ranges from 0 to 12 (high scores indicate poor oral health).

### Linguistic adaptation

We adhered to the guidelines provided by Beaton et al. [17] throughout the translation process. Forward translation from English to Hungarian was performed by two bilingual translators and consolidated by a third translator. Backward translation was performed by one native English speaker without access to the original questionnaire. After consolidation by the translator team and 15 clinicians, the draft instrument was piloted by 20 randomly selected patients via think-aloud interviews. As a result, we adjusted the polarity of items 3, 5, and 7, ensuring that a higher score for each answer now indicates fewer problems. The Hungarian GOHAI (GOHAI-HU) inquiries about problems in the last 3 months, and participants respond on a 5-point Likert-type frequency scale (1 = always; 2 = often; 3 = sometimes; 4 = seldom; 5 = never), resulting in ADD-GOHAI-HU scores ranging from 12 to 60. The stages of cross-cultural adaptation for the Hungarian GOHAI version are detailed in Appendix Fig. S1. The Hungarian and original English versions of GOHAI can be found in Appendix Table S1.

### Participants

**Fig. 1:**
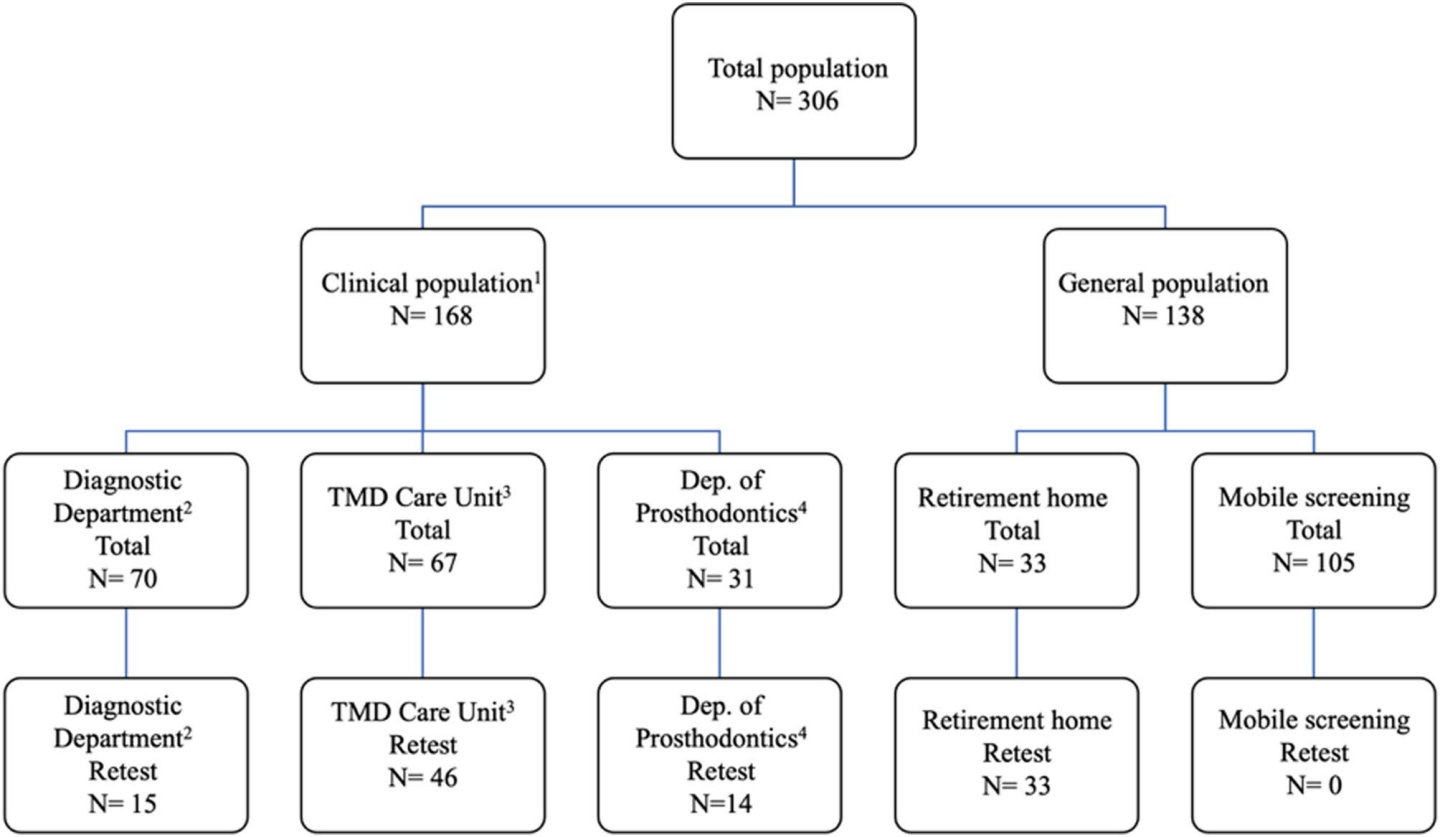
Study sample recruitment ^1^Clinical population from Semmelweis University, Faculty of Dentistry’s outpatient services: ^2^ Department of Oral Diagnostics, ^3^ Temporomandibular Disorders Care Unit, ^4^ Department of Prosthodontics

The research was approved by Semmelweis University Regional and Institutional Committee of Science and Research Ethics (permit number 61/2023), and all participants provided their informed consent before their enrolment in the study. The study sample comprised two groups: participants from the general population without primary dental concerns and clinical patients with primary dental issues. The general patient cohort was recruited in Budapest from a nursing home, and the general population attended mobile health screening kiosks on 06/05/2023 and 20/05/2023. Clinical patients were sourced from SU’s Department of Oral Diagnostics, Temporomandibular Disorders (TMD) Care Unit, and Department of Prosthodontics. All patients underwent examination by a dental medical doctor (KM or JO). For those patients whose stable oral health status was affirmed, the questionnaire was repeatedly administered a week after the initial interview by the same doctor personally or via phone. See Fig. 1 for study sample recruitment details.

The sample size was determined using the COSMIN modified GRADE approach, which recommends a total sample size exceeding 100 for high quality of evidence on hypothesis tests, the evaluation of test-retest reliability, and conducting CFA. We recruited a sample to meet at least 100 respondents in each subgroup, including clinical and general populations, age groups, and the retest sample.

### Data

The questionnaire had five main parts. First, basic demographic information was gathered. Second, participants answered questions about their oral health, including self-assessed oral health via Oral-Health Single Question (OH-SQ), dental treatment need (DTN), gingival bleeding, chewing ability, oral pain, dry mouth, halitosis, and satisfaction with the appearance of teeth. Third, participants completed the GOHAI-HU section. Fourth, they responded to the OHIP-14 section and the 5-level version of the EQ-5D general health-related quality of life (GHRQoL) instrument (EQ-5D-5L). The EQ-5D questionnaire consists of two parts: the descriptive system inquiries about the level of problems in five domains (i.e., mobility, self-care, usual activities, pain/discomfort, and anxiety/depression), while the EQ-VAS is a visual analogue scale inquiring the respondents’ subjective health status between the best (100) and worst (0) imaginable health [20].

Following the interviews, an oral examination was conducted. The examination included recording the number of teeth (up to 28), missing teeth, decayed teeth, filled/crowned teeth, mobile teeth, and the type of dentures worn (fixed/removable), along with any observed mucosal changes. In addition to the questions about GOHAI-HU and the persistence of symptoms asked during the first session, the second questionnaire includes two questions of opposite polarity concerning the stability of oral health.

### Statistical analysis

Incompletely filled questionnaires were excluded from the evaluation. For every psychometric property, we considered the terminology and reference values defined and proposed by COSMIN [14].

### Descriptive analyses

The proportion of female respondents, mean and standard deviation (SD) of age, ADD-GOHAI, SC-GOHAI, OHIP, EQ-5D-5L index, and EQ-VAS values were reported by each subsample. The proportions of respondents by the chosen level of each GOHAI item were tabulated. The floor and ceiling effect of ADD-GOHAI and SC-GOHAI was assessed against a 15% threshold [21]. Subgroups were compared using the independent t-test and two-sided significance test or one-way ANOVA in case of multiple categories.

### Content validity

Given the widespread use of GOHAI over decades in several countries, we considered its content validity as established [15]. Therefore, content validity was not assessed in this study.

### Internal structure and structural validity

When developing GOHAI, Atchison proposed a three-dimensional structure for it [5]. Following Kressin et al. [22], we classified the items into these three domains as follows: Physical function (items 1–4), Psychosocial function (items 6, 7, 9–11), and Pain and discomfort (items 5, 8, 12). A COSMIN systematic review showed that factor structure of the GOHAI exhibits significant variability. While studies identify structures of one to five factors, an overall unidimensional structure is more plausible [15].

We compared two Confirmatory Factor Analysis (CFA) models for ADD-GOHAI scores (Appendix Fig. S6). In the three-factor model, we allowed correlations between the “Physical function,” “Psychosocial function,” and “Pain and discomfort” factors, assuming that these three primary dimensions reflect a single underlying OHRQoL construct (i.e., a secondary factor). In contrast, the one-factor model assumed a single OHRQoL construct with correlated errors between items within each of the three factors. We also calculated modification indices to identify potential improvements in model fit. While both models reflect a similar structure, they differ in parameter constraints and degrees of freedom (i.e., the number of freely estimable parameters), resulting in different factor loadings [23, 24]. The adequacy of the CFA model was evaluated using the following fit indexes and threshold values: comparative fit index (CFI) > 0.95, standardized root-mean-square residual (SRMR) < 0.08, and root-mean-square error of approximation (RMSEA) < 0.06. Given the non-normal distribution, we applied the Satorra-Bentler estimator using a scaled chi^2^ statistic when calculating the fit-indexes [25]. By evaluating the modification indexes, we allowed minimal and justifiable amendments of the theoretical models to achieve better fit [26].

We also conducted exploratory factor analysis (EFA) for ADD-GOHAI. We computed varimax orthogonal rotated factor loadings. The number of extracted factors was determined by the Kaiser-Guttman criterion (i.e., factors with eigenvalues > 1 were retained), observing the elbow of the Scree plot, and the lowest Bayesian Information Criterion [27]. Factor loadings of 0.32 and 0.5 were considered as minimum and adequate / strong, respectively [28]. CFA and EFA were conducted on the total sample, general and clinical population subgroups, age subgroups, and retest data. On the total sample we also explored inter-item Spearman correlations.

### Internal consistency

To assess internal consistency, Cronbach’s alpha coefficient was calculated for the three dimensions of GOHAI, as well as for the entire questionnaire. Alpha values ≥ 0.7 were regarded as sufficient.

### Measurement invariance

We tested the measurement invariance of both ADD-GOHAI and SC-GOHAI using multi-group CFA by the clinical and general population subgroups, as well as the 18–64 years old and 64 + years old subgroups. Following the hierarchy of measurement invariance [29], first, we ensured configural invariance by checking the fit indexes of the one- and three-dimensional CFA models for each subgroup. We assumed that the same factor structure shows best fit in each subgroup. Configural invariance ensures the same factor structure across groups, indicating that respondents interpret the constructs similarly across groups. Then, metric invariance was tested for each GOHAI item via the chi^2^ test to compare the factor loadings by subgroups. Overall metric invariance was examined using a joint Lagrange multiplier (LM) test of all factor loadings. Metric invariance suggests that a change in item scores results in a similar change in the latent construct OHRQoL in the subgroups. Metric invariance suggests that factor loadings are consistent across groups, meaning that items reflect the latent construct (OHRQoL) to the same degree. Consequently, any differences in the latent construct are represented in similar item response patterns across groups. Strong invariance assuming equal factor loadings and intercepts was also tested by each item and overall. Strong invariance suggests that items contribute similarly to both the level and the change in OHRQoL across subgroups. With p values < 0.05, the hypothesis of equal factor loadings and equal intercepts can be rejected, suggesting that the contribution of items to the overall GOHAI score differs between subgroups. Strong invariance suggests that both factor loadings and item intercepts are consistent across groups. This ensures that differences between group mean scores reflect similar differences in the latent construct across groups rather than measurement bias. Sample heterogeneity beyond the tested subgroups (i.e., clinical heterogeneity within age groups, or the clinical or general population samples) may interfere with the testing of measurement invariance. Hence, our MI results have to be interpreted with caution.

### Reliability

For testing reliability, stable patient status and consistent test conditions are required between repeated measurements. Therefore, we administered two questions with opposing polarities to test whether participants’ oral health remained unchanged. The same interviewer recorded the questionnaire for the second time with a one-week interval. We measured test-retest reliability for ADD-GOHAI and SC-GOHAI through the intraclass correlation coefficient agreement formula (ICC model 2.1) [30]. For each participant, repeated administrations of GOHAI were performed by the same dental doctor, so the systematic difference between test-retest scores due to raters was negligible. ICC ≥ 0.7 was regarded as a threshold for sufficient test-retest reliability.

*Standard Error of Measurement (SEM) and smallest detectable change (SDC).*

Standard error of measurement (SEM) calculation was based on the SEM agreement formula [30, 31]:

1 $$\:SEM=\sqrt{{\sigma\:}_{rater}^{2}+{\sigma\:}_{residual}^{2}}$$

where $\:{\sigma\:}_{rater}^{2}$ is the variance due to raters (i.e., differences between test and retest administrations by the same doctor in this study) and $\:{\sigma\:}_{residual}^{2}$ is the residual error variance. When measuring GOHAI for a respondent, due to the measurement error, the true GOHAI score lies within the 95% confidence interval (95%CI) of the measured value calculated as$\:\:\pm\:1.96*SEM$. The smallest detectable change (SDC) (i.e., the change in the GOHAI score of a respondent, which is not attributable to measurement error) was calculated by the following formula:

2 $$\:SDC=1.96*\sqrt{2}*SEM$$.

ICC, SEM, 95%CI for true scores and SDC values were calculated for the total sample and each subgroup where test-retest scores were available.

### Construct validity

During the assessment of construct validity, we followed the expected effect size magnitudes and directions defined in a COSMIN review of GOHAI [32], and we followed Cohen’s criteria when interpreting the magnitude of effects [33]. Convergent validity was tested via Spearman correlation due to the non-normal distribution of GOHAI scores. We expected strong correlation (*r* > 0.50) between GOHAI and instruments measuring similar constructs (OHIP-14 for OHRQoL, and OH-SQ for self-assessed oral health). For instruments measuring related but dissimilar self-reported constructs (i.e., dental treatment need, EQ-5D-5L index, EQ-VAS), the expected correlation was moderate (*r* ~ 0.30–0.50). For physician-reported objective measures of dental status (e.g., DMFT or number of teeth), we expected weak (*r* ~ 0.10–0.30) correlation, as dental problems are usually alleviated by prosthodontic or dental treatments, and dental status does not reflect the entire spectrum of oral health.

We assessed discriminative (i.e., known group) validity using independent t-tests and one-sided p values. The effect size was reported as standardized mean difference. We hypothesized that patients with poor dental status, bleeding gum, oral pain, chewing problems, xerostomia, halitosis, and aesthetical problems would have worse GOHAI scores, and the magnitude of the effect size would be at least small. Altogether, for convergent validity we evaluated six hypotheses and for known-groups validity ten.

### Criterion validity and responsiveness

As for all PROMs, there is no available gold standard measure for OHRQoL instruments, so we did not investigate criterion validity (i.e., the agreement with a gold standard) in this study [34]. The responsiveness of GOHAI was not investigated either.

## Results

### Descriptives analyses

#### Sample characteristics

Altogether, complete data were available from 306 participants. In nine cases, data were incomplete, so these were excluded (9/315, 2.8%). The questionnaire was administered twice to 108 individuals. For the TMD population, the second interviews were conducted via telephone (*n* = 46), while for all other cases, they were conducted in person, in the same location (*n* = 62). Mean (SD) age of the total sample was 57.3 (20.4) years; 71.2% were female; 16.0%, 43.1%, and 40.9% had primary, secondary, and tertiary education respectively; 75.5% lived in cities, 16.7% in towns, and 7.8% in rural areas. Over half of the participants (54.9%) were recruited from clinical populations, and 45.1% were 65 + years old. Table 1. and Appendix Table S2. displays the descriptive statistics for each subgroup. As expected, the clinical population showed a lower average ADD-GOHAI score than the general population (t-test *p* = 0.002). The clinical population’s GHRQoL was somewhat better than the general population, which is explained by the age difference. The ADD-GOHAI scores did not differ between the younger and older age groups (t-test *p* = 0.127). Despite similar mean age, GOHAI scores of respondents with primary education showed worse OHRQoL than those with tertiary education (ANOVA *p* = 0.001). The ADD-GOHAI scores were similar for the urban and rural residents (ANOVA *p* = 0.150).

### Distribution of GOHAI scores

Appendix Fig S2 shows the distribution of ADD-GOHAI scores by subgroup, with the proportion of respondents scoring the highest and lowest possible scores. While no respondents scored 12 (worse OHRQoL), indicating a lack of floor effect, 18.8% in the total sample and 20.3% in the 65 + years old subgroup scored 60 (best OHRQoL), indicating that ADD-GOHAI has a ceiling effect. Appendix Fig. S3 shows the distribution of SC-GOHAI scores by subgroup. In the total sample and all subgroups, over 15% of respondents had a 0 score (best OHRQoL), indicating a floor effect for SC-GOHAI. Due to a greater proportion of respondents without problems in OHRQoL, both ADD-GOHAI and SC-GOHAI have skewed distribution.

### Responses by item

**Fig. 2:**
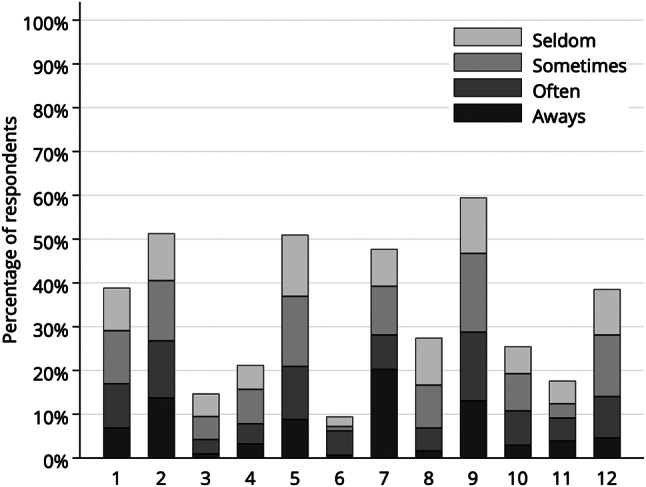
The frequency distribution of problems by the items of GOHAI

Most respondents (59.5%) indicated the presence of problems on item 9 (worry about the problems with teeth, gums, or dentures), while problems occurred least often (9.5%) on item 9 (limiting contacts with people due to the condition of teeth or dentures). On item 7 (problems with the looks of teeth, gums, or dentures), 20.3% of respondents always had problems, while on item 6, only 0.7% indicated the always option. The proportion of responses by item is shown in Fig. 2; further details are provided in the Appendix (Table S3). Furthermore, the proportion of sometimes / often / always responses contributing to SC-GOHAI is provided in the Appendix (Fig. S4).

**Table 1:** Sample characteristics and Mean values with standard deviations for Add-GOHAI, SC-GOHAI, OHIP, EQ-5D-5L, and EQ-VAS by subgroups

	Population	Site	*N*	Female %	Age mean (SD) years	ADD-GOHAI mean (SD)	SC-GOHAI mean (SD)	OHIP mean (SD)	EQ-5D-5L index mean (SD)	EQ VAS mean (SD)
Retest No	General	Screening kiosk	105	67.6%	61.3 (15.6)	51 (8.7)	2.7 (2.6)	65.6 (6.9)	0.86 (0.23)	69.9 (17.7)
	Clinical	SU-OD^a^	55	67.3%	48.9 (19.3)	48.5 (9.1)	3.6 (2.8)	62.1 (9.6)	0.91 (0.13)	70.5 (17.9)
		SU-DP^b^	17	76.5%	61.7 (17.4)	46.4 (7.9)	4.5 (2.3)	61.9 (8.1)	0.87 (0.13)	73.2 (16)
		SU-TMD^c^	21	90.5%	43.9 (19.4)	49.2 (8.6)	3.4 (2.6)	61.5 (9.2)	0.9 (0.14)	79 (12)
	Subtotal	-	198	70.7%	56 (18.5)	49.7 (8.8)	3.2 (2.7)	63.9 (8.2)	0.88 (0.19)	71.3 (17.2)
Retest Yes	General	Retirement home	33	69.7%	87.8 (4.4)	55.4 (5.9)	1.4 (1.9)	67.4 (3.8)	0.76 (0.29)	67.6 (20.6)
	Clinical	SU-OD^a^	15	60%	49.7 (18.8)	51.9 (7.5)	2.7 (2.7)	62.8 (10.5)	0.93 (0.12)	77.9 (19.4)
		SU-DP^b^	14	92.9%	62.4 (11.9)	49.4 (9.1)	3.3 (2.8)	62.4 (10)	0.93 (0.07)	73.6 (16.2)
		SU-TMD^c^	46	71.7%	42.1 (14.6)	49.2 (8.7)	3.5 (2.8)	60.8 (9.9)	0.91 (0.15)	74.3 (16.6)
	Subtotal	-	108	72.2%	59.8 (23.4)	51.5 (8.2)	2.7 (2.7)	63.3 (9)	0.87 (0.2)	72.6 (18.4)
Population	General	-	138	68.1%	67.6 (17.9)	52.1 (8.3)	2.4 (2.5)	66 (6.3)	0.84 (0.25)	69.4 (18.4)
	Clinical	-	168	73.8%	48.9 (18.4)	48.9 (8.6)	3.5 (2.7)	61.8 (9.5)	0.91 (0.13)	73.8 (16.7)
Age-group	Age:18–64	-	168	70.8%	41.8 (12.9)	49.7 (8.1)	3.3 (2.6)	63.1 (8.7)	0.92 (0.14)	75.7 (15.9)
	Age: 64 +	-	138	71.7%	76.2 (8.2)	51.2 (9.2)	2.7 (2.7)	64.5 (8.1)	0.82 (0.23)	67.1 (18.5)
Education	Primary	-	49	63.3%	56.6 (19.6)	46.8 (10.9)	4.1 (3.2)	60.5 (11.3)	0.83 (0.22)	66.4 (19.4)
	Secondary	-	132	80.3%	57.7 (20.3)	50.2 (8.5)	3.0 (2.6)	63.3 (8.6)	0.87 (0.2)	70.7 (17.5)
	Tertiary	-	125	64.8%	57.3 (21)	51.9 (7.3)	2.5 (2.4)	65.3 (6.5)	0.90 (0.17)	75.1 (16.4)
Population	City	-	231	70.6%	59.6 (20.5)	50.9 (8.6)	2.8 (2.7)	64.3 (8.1)	0.87 (0.19)	70.8 (17.2)
	Town	-	51	78.4%	50.0 (18)	48.9 (8.1)	3.5 (2.6)	62.1 (8.9)	0.9 (0.16)	75.9 (17.7)
	Rural area	-	24	62.5%	51.4 (19.9)	48.2 (10.1)	3.8 (3)	61.2 (10.3)	0.81 (0.27)	72.3 (20.3)
**Total**	**-**	**-**	**306**	**71.2%**	**57.3 (20.4)**	**50.3 (8.6)**	**3 (2.7)**	**63.7 (8.5)**	**0.87 (0.19)**	**71.8 (17.6)**

^a^ Semmelweis University, Department of Oral Diagnostics; ^b^ Semmelweis University, Department of Prosthodontics; ^c^ Semmelweis University, Temporomandibular Disorders Care Unit

### Internal structure

#### Structural validity

Most inter-item correlations were weak or negligible, and only 23 out of 66 (34.8%) inter-item correlations were moderate or strong (Appendix Fig. S5).

Contrary to the three-factor model, the single-factor model showed good fit across all subgroups for both ADD-GOHAI and SC-GOHAI, with adequate internal consistency (Table 2). The loadings on the main OHRQoL factor in both CFA models and the EFA model followed a similar pattern, with only minor differences (Appendix Fig. S7). All items but 4, 6, and 12 had at least minimal loading on the single factor in both the total sample and retest data (Appendix Fig. S8). The one-factor model met all good fit criteria of COSMIN in the total sample, the clinical subsample, and both age groups. Also, the one-factor model showed acceptable fit in all criteria on the retest data. In addition to the correlated errors between the items of the three proposed factors, we allowed error correlation between items 2 (trouble with biting or chewing) and 3 (problems with swallowing) as well as items 3 and 5 (eating with discomfort). Although these items are from the domains of “physical function” and “pain and discomfort”, they are all conceptually related to problems with eating, which justifies the amendment of the model. In EFA, the eigenvalues and Scree plot suggested a single factor structure, while BIC suggested a three and two factor solution in the total sample and retest data, respectively (Appendix Fig. S9, S10). In the nonrotated solution, the first factor explained 85.4% and 81.1% of the variance in the total sample and retest data, respectively. In the rotated solution in both datasets, items 1, 2, and 5 (problems with eating) had high loadings on the main factor, and items 6, 10, and 11 (psychosocial problems) had high loadings on the minor second factor while the loadings on a minor third factor were mostly weak and inconsistent. Several items had high loadings on multiple factors, and the theoretical pain/discomfort domain did not emerge as an independent dimension (Appendix Fig. S11-S14). Altogether, the single factor structure shown by both CFA and EFA analyses supports the use of a single OHRQoL score instead of forming subscales for various OHRQoL domains.

### Internal consistency

The Cronbach values for the total model and each subgroup also indicated better internal consistency for a single-factor model for both ADD-GOHAI and SC-GOHAI (Table 2). In all subgroups, the single-factor model had adequate internal consistency with values greater than 0.70.

### Measurement invariance

Configural invariance for ADD-GOHAI and SC-GOHA could be concluded because the single-factor structure best fits all studied subgroups.

For ADD-GOHAI, the overall LM test showed significant overall difference between the factor loadings of the clinical and general populations, with the largest difference between items 11 (feeling uncomfortable with eating in front of others) and 12 (sensitivity of teeth or gums to hot, cold or sweets). However, there was no overall difference in the factor loadings by age group. The intercepts differed between the clinical and general populations and the subgroups by age. For SC-GOHAI, the overall difference between factor loadings was not significant, but item 12 differed between the clinical and general populations, and item 11 between age groups. The overall difference between intercepts was significant for both subgroups.

Altogether, strong measurement invariance could not be demonstrated for ADD-GOHAI or SC-GOHAI, suggesting that mean score differences across groups may be due to measurement bias and do not necessarily reflect true differences in OHrQoL. However, SC-GOHAI showed metric invariance between clinical and general populations as well as age groups, indicating that changes or differences in SC-GOHAI scores reflect similar differences in the latent construct (OHRQoL) across these subgroups. For ADD-GOHAI, metric invariance was shown only across age groups, but not between general and clinical populations. The potentially different measurement properties of ADD-GOHAI should be considered when comparing or synthesizing results across subpopulations. For instance, similar effect sizes may reflect different changes in OHRQoL in different populations. Additionally, associations between ADD-GOHAI scores and other variables may vary between populations due to these measurement differences rather than true differences in the underlying constructs. Since SC-GOHAI demonstrated metric invariance across clinical and general populations and age groups, it is a suitable tool for tracking changes within these groups and for assessing the relationship of OHRQoL with other variables.

**Table 2:** Fit indexes of the CFA of the three-factor and one-factor model and cronbach 𝛼 values

	Model	Subgroup	n	CFI^a^	TLI^b^	GFI^c^	RMSEA^d^	SRMR^e^	Total	Phys^g^	Psy^h^	PD^i^	Measurement invariance	
									α^f^	α	α	α	Loadings^j^	Intercepts^k^
ADD-GOHAI	Three-factor	Total	306	0.93	0.91	0.87	0.059	0.062	-	0.70	0.74	0.50	-	-
		Retest	108	0.89	0.86	0.77	0.077	0.086	-	0.65	0.77	0.42	-	-
		General	138	0.85	0.81	0.75	0.084	0.103		0.73	0.70	0.47	-	-
		Clinical	168	0.93	0.91	0.84	0.064	0.063	-	0.68	0.76	0.33	-	-
		18–64 years old	168	0.93	0.91	0.83	0.058	0.073	-	0.70	0.70	0.41	-	-
		64 + years old	138	0.95	0.93	0.84	0.053	0.071	-	0.70	0.78	0.56	-	-
	One-factor with correlated errors	Total	306	0.98	0.95	0.94	0.042	0.041	0.83	-	-	-	-	-
		Retest	108	0.96	0.92	0.88	0.059	0.068	0.84	-	-	-	-	-
		General	138	0.95	0.89	0.87	0.062	0.070	0.83	-	-	-	0.002	0.003
		Clinical	168	0.99	0.97	0.93	0.036	0.039	0.82	-	-	-		
		18–64 years old	168	0.97	0.95	0.91	0.068	0.051	0.81	-	-	-	0.303	< 0.001
		64 + years old	138	0.97	0.95	0.90	0.047	0.050	0.85	-	-	-		
SC-GOHAI	Three-factor	Total	306	0.93	0.91	0.86	0.055	0.061	-	0.63	0.68	0.46	-	-
		Retest	108	0.90	0.88	0.76	0.066	0.081	-	0.60	0.70	0.45	-	-
		General	138	0.82	0.76	0.71	0.90	0.110	-	0.65	0.66	0.57	-	-
		Clinical	168	0.91	0.88	0.80	0.062	0.065	-	0.62	0.68	0.34	-	-
		18–64 years old	168	0.86	0.82	0.76	0.076	0.081	-	0.64	0.65	0.39	-	-
		64 + years old	138	0.91	0.88	0.78	0.063	0.079	-	0.62	0.71	0.49	-	-
	One-factor with correlated errors	Total	306	0.99	0.99	0.95	0.022	0.035	0.78	-	-	-	-	-
		Retest	108	0.95	0.91	0.86	0.058	0.068	0.80	-	-	-	-	-
		General	138	0.93	0.87	0.86	0.067	0.065	0.79	-	-	-	0.115	0.026
		Clinical	168	0.98	0.96	0.91	0.037	0.040	0.76	-	-	-		
		18–64 years old	168	0.97	0.94	0.90	0.045	0.051	0.76	-	-	-	0.142	< 0.001
		64 + years old	138	0.96	0.99	0.90	0.021	0.049	0.81	-	-	-		

^a^Comparative fit index; ^b^Tucker-Lewis index; ^c^Goodness of Fit Index; ^d^Root Mean Square Error of Approximation; ^e^Standardized Root Mean Residuals; ^f^Cronbach alpha, ^g^Physical function: items 1–4; ^h^Psychosocial function: items 6, 7, 9–11, ^i^Pain and discomfort: items 5, 8, 12. ^j^LM test p value for equal factor loadings; ^k^ LM test p value for equal intercepts

### Remaining measurement properties

#### Reliability and measurement error

The test-retest reliability of both ADD-GOHAI and SC-GOHAI was adequate in the total sample as well as all subgroups with ICC values ranging between 0.87 and 0.96 (Table 3). The smallest detectable change is approximately 5 points using ADD-GOHAI and 2–3 points using SC-GOHAI. Compared to the measurement range, ADD-GOHAI scores are able to detect more nuanced change in individual patients’ OHRQoL than SC-GOHAI. However, when using ADD-GOHAI for individual follow-up, users should be aware that, despite a score range of 48 points, a minimum change of 5 points is required to indicate a true change in an individual’s OHRQoL, while smaller differences may be indistinguishable from measurement error.

**Table 3:** Reliability and measurement error in subsamples with repeat measurements

Score	Sample	*N*	ICC	SEM	±95%CI of true score	SDC
ADD-GOHAI	Total	108	0.95	1.8	±3.6	5.1
	General	33	0.89	1.9	±3.7	5.2
	Clinical	75	0.96	1.8	±3.5	5.0
	18–64 years old	59	0.95	1.8	±3.6	5.1
	64 + years old	49	0.94	1.8	±3.5	5.0
SC-GOHAI	Total	108	0.91	0.8	±1.6	2.2
	General	33	0.87	0.6	±1.2	1.7
	Clinical	75	0.91	0.9	±1.7	2.4
	18–64 years old	59	0.90	0.9	±1.8	2.6
	64 + years old	49	0.93	0.6	±1.2	1.7

### Construct validity

Four out of the six hypotheses we tested for convergent validity supported the predefined criteria. As expected, the related constructs (OHIP-14, OH-SQ) showed a strong effect size correlation with ADD-GOHAI scores, while weakly related constructs such as general health showed small correlations (Visual Analog Scale (VAS) of EQ-5D-5L) and medium correlations (EQ-5D-5L score). The DMFT index and the number of teeth had negligible connection with GOHAI scores. This result is consistent with international data. This may be because correctly replaced missing teeth and well-filled teeth are perceived similarly to original teeth, and OHRQoL has a much more complex underlying construct than dental status.

All ten hypotheses we used to assess known-groups validity confirmed the predefined criteria. In five cases, we found a weak to moderate effect size (d = 0.2–0.5) with factors such as mobile teeth, mucosal lesions, bleeding gums, and specific populations. Halitosis, chewing problems, pain, DTN, and aesthetic dissatisfaction had a strong effect size (d > 0.5) suggesting that these observable signs or symptoms have greatest impact on QHRQoL. For detailed information about the results of hypotheses testing for construct validity see Table 4.

**Table 4:** Summary of results of hypotheses testing for convergent and known-groups validity

	Type of effect size	Variable	Effect size	*P* value^a^	Expected magnitude / sign	Hypothesis confirmed
Convergent validity	Spearman correlation	OHIP-14	0.84	< 0.001	> 0.50 / +	Yes
		OH-SQ	-0.52	< 0 0.001	> 0.50 / -	Yes
		EQ-5D-5L index	0.31	< 0.001	~ 0.30–0.50 / +	Yes
		EQ VAS	0.28	< 0.001	~ 0.30–0.50 / +	Yes
		DMFT	-0.08	0.143	~ 0.10–0.30 / -	No
		Number of teeth	0.08	0.169	~ 0.10–0.30 / +	No
Known- groups validity	Standardized mean difference	Mobile teeth (yes / no)	-0.30	0.066	> 0.10 / -	Yes
		Mucosal lesion (yes / no)	-0.49	0.001	> 0.10 / -	Yes
		Gum bleeding (yes / no)	-0.22	0.048	> 0.10 / -	Yes
		Chewing problem (yes / no)	-1.35	< 0.001	> 0.10 / -	Yes
		Pain (yes / no)	-0.72	< 0.001	> 0.10 / -	Yes
		Xerostomia (yes / no)	-0.45	< 0.001	> 0.10 / -	Yes
		Halitosis (yes / no)	-0.68	< 0.001	> 0.10 / -	Yes
		Aesthetic satisfaction (yes / no)	1.02	< 0.001	> 0.10 / +	Yes
		Dental treatment need (yes / no)	-0.94	< 0.001	> 0.10 / -	Yes
		Populations (clinical / general)	-0.37	< 0.001	> 0.10 / -	Yes

^a^For known-groups validity: independent t-test with one-sided p-value

## Discussion

In this study, we developed the Hungarian version of GOHAI and used the COSMIN guidelines innovatively for a linguistic adaptation of an OHRQoL instrument. We also examined the SEM, SDC, and MI of GOHAI for the first time.

Construct validity is how well a PROM reflects the underlying concept. Based on hypothesis testing, GOHAI effectively measures OHRQoL and accurately reflects Locker’s comprehensive conceptual model [35]. Although Locker previously questioned the relationship between GOHAI and GHRQoL [36], literature data indicate a clear association [15]. Out of 24 studies, 23 found at least a weak correlation, consistent with our findings, suggesting that OHRQoL is part of GHRQoL. Perceptions of dental status are highly subjective. In the literature, approximately 28% of the hypotheses related to dental status did not find any correlation with ADD-GOHAI scores [15]. The weak, often undetectable, or even inverse relationships observed with objectively measurable oral factors highlight the significance of PROMs in dental research. PROMs are essential for assessing patients’ subjective experiences, which cannot be fully captured by easily measurable objective indicators alone.

In our study, ADD-GOHAI exhibited a significant ceiling effect, while SC-GOHAI showed a floor effect. Hassel’s study reported that 7.1% of the population achieved the highest score [10], while Locker’s study showed an 8.4% rate [37]. In contrast, our study’s 18.8% rate possibly reflects better socio-demographic characteristics of the participants. Altogether, GOHAI has a limited score range to capture differences in individuals with better-than-average OHRQoL, while it has a wide score range to measure poor OHRQoL. The properties of the widely used GHRQoL measures, such as EQ-5D, are similar [38].

The factor structure of the GOHAI exhibits significant variability, with the number of identified factors ranging from one to five [15]. This inconsistency may be attributed not only to differences in population characteristics but also to the structure of the questionnaire itself. Our findings, consistent with international literature, support a single-factor model [5, 39]. However, the single-factor structure should be confirmed in further samples. This structure suggests that the GOHAI items are closely related and may not be separable into distinct OHRQoL domains. While GOHAI’s primary aim is to assess the single underlying construct of OHRQoL reflectively, it also addresses diverse and independent oral conditions that affect OHRQoL. Hence, GOHAI is a mixed reflective and formative instrument [16], which may explain inconsistencies in its internal structure and lack of measurement invariance between different populations. Furthermore, GOHAI lacks sufficient indicators to represent orofacial appearance, an increasingly accepted fourth dimension of OHRQoL [40]. Our factor analysis showed a dominant factor strongly loading on items describing eating problems and a secondary factor loading on items of psychosocial problems, while pain/discomfort or appearance did not emerge as salient dimensions.

Consistent with a meta-analysis finding of a 0.81 Cronbach’s 𝛼 for ADD-GOHAI, our study showed a strong internal consistency with a Cronbach’s 𝛼 of 0.83 [15].

The strength of our study is that on a relatively large and diverse sample, MI, SEM and SDC were assessed.

While configural invariance was observed across subgroups for both ADD-GOHAI and SC-GOHAI, strong measurement invariance was not achieved. Additionally, ADD-GOHAI lacked metric invariance between the general and clinical populations. Assessment of OHRQoL is highly subjective and can vary culturally, by gender, and across age groups. While an aesthetic issue may severely impact QoL for younger individuals, it can be completely accepted in older age, often without negative consequences. In older adults, habituation and changing expectations can occur, making direct comparisons between the two groups potentially misleading. Our results support this observation. Additionally, differences between clinical and general populations highlight that the experience of oral health may fundamentally differ between the two groups. SC-GOHAI was more stable between subgroups. We note that sample heterogeneity beyond the tested subgroups (i.e., clinical heterogeneity within age groups or the clinical or general population samples) may interfere with the testing of measurement invariance. Hence, our MI results have to be interpreted with caution.

The questionnaire’s suitability for individual follow-up is questionable, especially due to the relatively high SDC thresholds—approximately 5 points for ADD-GOHAI and 2–3 points for SC-GOHAI. While ADD-GOHAI appears more appropriate for personal monitoring when comparing these scoring methods, any observed changes must be considered in light of the measurement error associated with GOHAI. However, the responsiveness of GOHAI scores remains an area for further research. For the full 49-item OHIP, the minimal important difference (i.e., the smallest score difference perceivable by patients) is 6 points [41]. The smallest detectable change (SDC), in contrast, reflects measurement error (i.e., the change in an individual’s score that reflects real changes and not mere chance). Users should be aware that despite the wide score range of 48 between 12 and 60 points, at least a 5-point change is needed to signify a real change in an individual’s OHRQoL status.

Some limitations of this study should be acknowledged. The predominantly urban and higher-educated participant sample may affect generalizability [42]. Future research should explore the validity of the GOHAI in diverse socioeconomic contexts and cultural settings.

## Conclusion

The Hungarian version of the GOHAI demonstrates satisfactory psychometric properties across both general and clinical populations, as well as among younger and older age groups. While it serves as a valuable tool for measuring OHRQoL, its limitations—particularly regarding responsiveness and individual follow-up—should be acknowledged. Our findings suggest that in the era of personalized dentistry, there is a need for more sensitive PROMs with enhanced measurement properties. Future studies should refine the GOHAI to improve comparability across diverse populations and address the ceiling effect, which limits its ability to detect subtle changes in individuals with good overall oral health. Additionally, it is crucial to adapt the GOHAI or develop new instruments tailored to specific subgroups, such as individuals with chronic dental conditions. This limitation should be considered regarding GOHAI’s clinical use, especially concerning nuanced changes in oral health.

## Electronic supplementary material

Below is the link to the electronic supplementary material.

Supplementary Material 1

## Data Availability

The datasets used and analysed during the current study are available from the corresponding author on reasonable request.

## Abbreviations

dPROs: Dental Patient Reported Outcomes
OHRQoL: Oral Health-Related Quality of Life
OHIP: Oral Health Impact Profile
GOHAI: General Oral Health Assessment Index
PROMs: Patient Reported Outcome Measures
COSMIN: Consensus-based Standards for the selection of health Measurement INstruments
SEM: Standard Error of Measurement
SDC: Smallest Detectable Change
MI: Measurement Invariance
CTT: Classical Test Theory
ADD-GOHAI: Additive Score of General Oral Health Assessment Index
SC-GOHAI: Simple Count Score of General Oral Health Assessment Index
GOHAI-HU: Hungarian Version of the General Oral Health Assessment Index
OH-SQ: Oral-Health Single Question
DTN: Dental Treatment Need
TMD: Temporomandibular Disorders
GHRQoL: General Health-Related Quality of Life
EQ-5D-5L: 5-Level Version of the EQ-5D General Health-Related Quality of Life Instrument
EQ-VAS: EQ-5D Visual Analogue Scale
CFA: Confirmatory Factor Analysis
CFI: Comparative Fit Index
SRMR: Standardized Root-Mean-Square Residual
RMSEA: Root-Mean-Square Error of Approximation
ICC: Intraclass Correlation Coefficient
LM: Lagrange Multiplier
VAS: Visual Analog Scale
DMFT: Decayed, Missing, and Filled Teeth Index
