# Supplementary material for: Validation of the Hungarian version of the General Oral Health Assessment Index (GOHAI) in clinical and general populations
Source: BMC Oral Health. 2024 Nov 19;24:1402. doi: 10.1186/s12903-024-05198-2 (PMC11575072; doi:10.1186/s12903-024-05198-2)

# Appendix

***Fig. S1. Stages of cross-cultural adaptation^1^***

*^1^Beaton DE, Bombardier C, Guillemin F, Ferraz MB. Guidelines for the process of cross-cultural adaptation of self-report measures. Spine (Phila Pa 1976). 2000;25(24):3186-91.*

***Table S1.: The Hungarian General Oral Health Assessment Index (GOHAI-HU)***

|  | **Az elmúlt három hónap során milyen gyakran fordult elő^1^,** | *Original, English version* | **Mindig** | **Gyakran** | **Néha** | **Ritkán** | **Soha^2^** |
| --- | --- | --- | --- | --- | --- | --- | --- |
| 1. | … hogy a fogaival, fogpótlásával vagy műfogsorával kapcsolatos problémák miatt kevesebbet evett, vagy másfajta ételeket választott? | *How often did you limit the kinds or amounts of food you eat because of problems with your teeth or dentures?* |  |  |  |  |  |
| 2. | … hogy nehézséget okozott valamilyen típusú étel harapása vagy rágása? (például rostos húsok vagy alma) | *How often have you trouble biting or chewing any kinds of food, such as firm meat or apples?* |  |  |  |  |  |
| 3. | … hogy nehezen nyelt? | *How often were you able to swallow comfortably?* |  |  |  |  |  |
| 4. | … hogy fogai, fogpótlása vagy műfogsora miatt nem tudta úgy kiejteni a szavakat, ahogy szeretné? | *How often have your teeth or dentures prevented you from speaking the way you wanted?* |  |  |  |  |  |
| 5. | … hogy kellemetlenséggel járt az evés? | *How often were you able to eat anything without feeling discomfort?* |  |  |  |  |  |
| 6. | … hogy fogai, fogpótlása vagy műfogsora állapota miatt kevesebbet találkozott másokkal? | *How often did you limit contacts with people because of the condition of your teeth or dentures?* |  |  |  |  |  |
| 7. | … hogy elégedetlen volt fogai, ínye, fogpótlása vagy műfogsora kinézetével? | *How often were you pleased or happy with the looks of your teeth and gums, or dentures?* |  |  |  |  |  |
| 8. | … hogy a szájában vagy szája környékén érzett fájdalom vagy kellemetlenség enyhítésére gyógyszert használt? | *How often did you use medication to relieve pain or discomfort from around your mouth?* |  |  |  |  |  |
| 9. | … hogy aggódott a fogai, ínye, fogpótlása vagy műfogsora állapota miatt? | *How often were you worried or concerned about the problems of your teeth, gums or dentures?* |  |  |  |  |  |
| 10. | … hogy zavarba jött vagy kellemetlenül érezte magát fogai, ínye, fogpótlása vagy műfogsora állapota miatt? | *How often did you feel nervous or self-conscious because of problems with your teeth, gums, or dentures?* |  |  |  |  |  |
| 11. | … hogy fogai, fogpótlása vagy műfogsora miatt kellemetlennek érezte, hogy mások jelenlétében egyen? | *How often did you feel uncomfortable eating in front of people because of problems with your teeth or dentures?* |  |  |  |  |  |
| 12. | ... hogy fogai vagy ínye érzékenyek voltak melegre, hidegre vagy édesre? | *How often were your teeth or gums sensitive to hot, cold, or sweets?* |  |  |  |  |  |

^1^ Over the past three months, how often has it occurred?

^2^ Always, Often, Sometimes, Seldom, Never

***Table S2. Descriptive statistics: median and IQR values of PROM scales***

|  | Population | Site | N | Female | Age | ADD-GOHAI | SC-GOHAI | OHIP | EQ-5D-5L index | EQ VAS |
| --- | --- | --- | --- | --- | --- | --- | --- | --- | --- | --- |
|  |  |  |  | % | mean (SD) years | median (IQR) | median (IQR) | median (IQR) | median (IQR) | median (IQR) |
| Retest No | General | Screening kiosk | 105 | 67.60% | 61.3 (15.6) | 53 (47-58) | 2 (1-4) | 69 (64-70) | 0.93 (0.84-1) | 75 (60-80) |
|  | Clinical | SU-OD^a^ | 55 | 67.30% | 48.9 (19.3) | 50 (45-55) | 3 (2-5) | 65 (62-68) | 0.96 (0.87-1) | 75 (60-80) |
|  |  | SU-DP^b^ | 17 | 76.50% | 61.7 (17.4) | 48 (41-52) | 5 (3-6) | 65 (57-69) | 0.87 (0.76-1) | 80 (70-80) |
|  |  | SU-TMD^c^ | 21 | 90.50% | 43.9 (19.4) | 53 (45-54) | 3 (2-5) | 65 (60-68) | 0.96 (0.86-0.96) | 80 (70-90) |
|  | Subtotal | - | 198 | 70.70% | 56 (18.5) | 52 (45-56) | 3 (1-5) | 67 (62-70) | 0.94 (0.86-1) | 75 (60-80) |
| Retest Yes | General | Retirement home | 33 | 69.70% | 87.8 (4.4) | 58 (53-60) | 1 (0-2) | 69 (66-70) | 0.91 (0.54-0.97) | 70 (50-90) |
|  | Clinical | SU-OD^a^ | 15 | 60% | 49.7 (18.8) | 54 (43-58) | 2 (1-6) | 68 (56-70) | 1 (0.93-1) | 85 (68-95) |
|  |  | SU-DP^b^ | 14 | 92.90% | 62.4 (11.9) | 51.5 (41-57) | 2.5 (1-6) | 66.5 (59-70) | 0.96 (0.87-1) | 77.5 (60-90) |
|  |  | SU-TMD^c^ | 46 | 71.70% | 42.1 (14.6) | 50.5 (45-56) | 3 (1-5) | 64 (55-69) | 0.96 (0.88-1) | 80 (70-85) |
|  | Subtotal | - | 108 | 72.20% | 59.8 (23.4) | 54 (47.5-58) | 2 (0-4.5) | 67 (60.5-70) | 0.96 (0.85-1) | 76.5 (55-90) |
| Population | General | - | 138 | 68.10% | 67.6 (17.9) | 54.5 (48-59) | 1.5 (0-4) | 69 (65-70) | 0.92 (0.83-1) | 72.5 (50-80) |
|  | Clinical | - | 168 | 73.80% | 48.9 (18.4) | 50.5 (44-56) | 3 (1-5) | 65 (59-69) | 0.96 (0.87-1) | 80 (65-85) |
| Age-group | Age:18-64 | - | 168 | 70.80% | 41.8 (12.9) | 52 (45-56) | 3 (1-5) | 66 (61-70) | 0.96 (0.89-1) | 80 (70-86) |
|  | Age: 64 + | - | 138 | 71.70% | 76.2 (8.2) | 53.5 (47-59) | 2 (0-5) | 68 (63-70) | 0.91 (0.76-1) | 70 (50-80) |
| Education | Primary | - | 49 | 63.30% | 56.6 (19.6) | 49 (40-58) | 4 (1-7) | 65 (57-70) | 0.92 (0.76-0.97) | 70 (50-80) |
|  | Secondary | - | 132 | 80.30% | 57.7 (20.3) | 52 (46-57) | 2 (1-5) | 67 (62-69.5) | 0.93 (0.85-1) | 75 (51-80) |
|  | Tertiary | - | 125 | 64.80% | 57.3 (21) | 54 (49-58) | 2 (0-4) | 68 (64-70) | 0.96 (0.88-1) | 80 (70-85) |
| Residence | City | - | 231 | 70.60% | 59.6 (20.5) | 53 (46-58) | 2 (0-5) | 68 (63-70) | 0.96 (0.86-1) | 75 (60-80) |
|  | Town | - | 51 | 78.40% | 50.0 (18) | 51 (46-55) | 3 (1-5) | 65 (59-68) | 0.96 (0.88-1) | 80 (70-90) |
|  | Rural area | - | 24 | 62.50% | 51.4 (19.9) | 52 (43-56) | 3 (2-6) | 65 (59-70) | 0.92 (0.7-1) | 70 (69-87) |
| **Total** | **-** | **-** | **306** | **71.20%** | **57.3 (20.4)** | **52.5 (46-57)** | **2 (1-5)** | **67 (62-70)** | **0.96 (0.86-1)** | **75 (60-85)** |

^a^ Semmelweis University, Department of Oral Diagnostics; ^b^ Semmelweis University, Department of Prosthodontics; ^c^ Semmelweis University, Temporomandibular Disorders Care Unit

Fig. S2. Distribution of ADD-GOHAI scores per subgroup


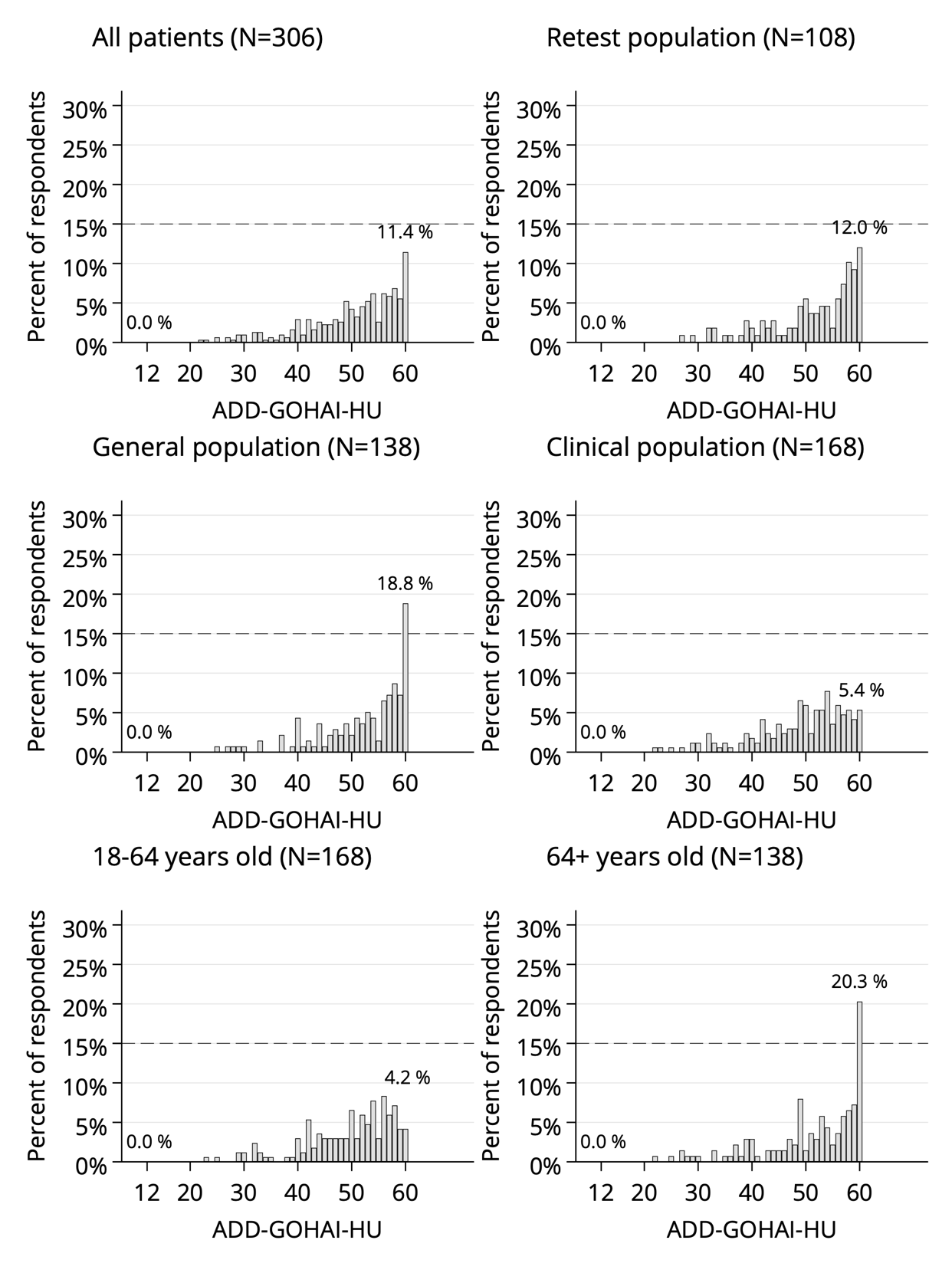


Fig. S3. Distribution of SC-GOHAI scores per subgroup


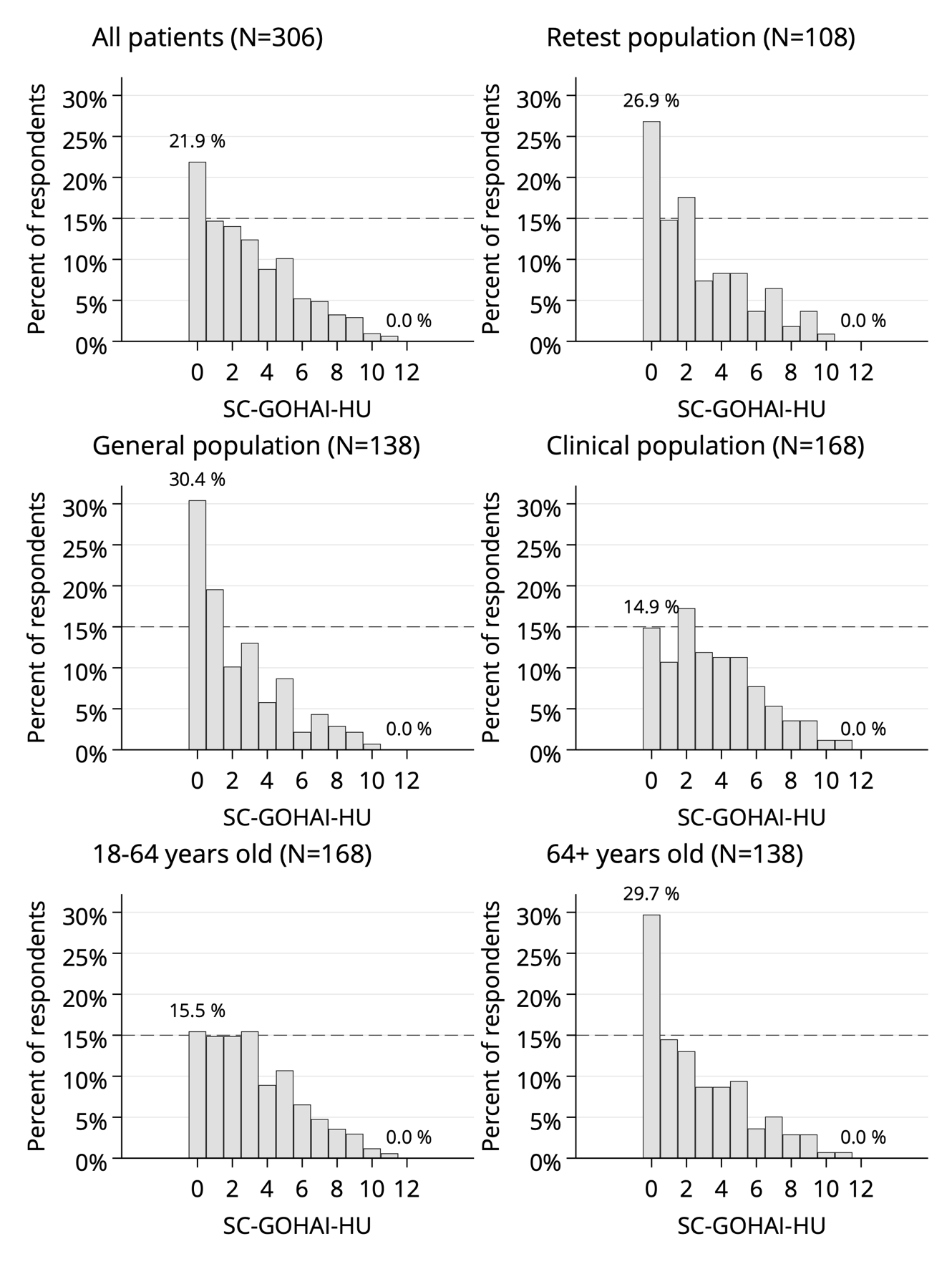


Table S3. The answer proportions of the GOHAI items

|  | 1 | 2 | 3 | 4 | 5 | 6 | 7 | 8 | 9 | 10 | 11 | 12 | TOTAL |
| --- | --- | --- | --- | --- | --- | --- | --- | --- | --- | --- | --- | --- | --- |
| Always | 6.9% | 13.7% | 1.0% | 3.3% | 8.8% | 0.7% | 20.3% | 1.6% | 13.1% | 2.9% | 3.9% | 4.6% | 6.7% |
| Often | 10.1% | 13.1% | 3.3% | 4.6% | 12.1% | 5.6% | 7.8% | 5.2% | 15.7% | 7.8% | 5.2% | 9.5% | 8.3% |
| Sometimes | 12.1% | 13.7% | 5.2% | 7.8% | 16.0% | 1.0% | 11.1% | 9.8% | 18.0% | 8.5% | 3.3% | 14.1% | 10.0% |
| Seldom | 9.8% | 10.8% | 5.2% | 5.6% | 14.1% | 2.3% | 8.5% | 10.8% | 12.7% | 6.2% | 5.2% | 10.5% | 8.5% |
| Never | 61.1% | 48.7% | 85.3% | 78.8% | 49.0% | 90.5% | 52.3% | 72.5% | 40.5% | 74.5% | 82.4% | 61.4% | 66.4% |

Fig. S4. The frequency distribution of sometimes / often / always responses adding up to SC-GOHAI
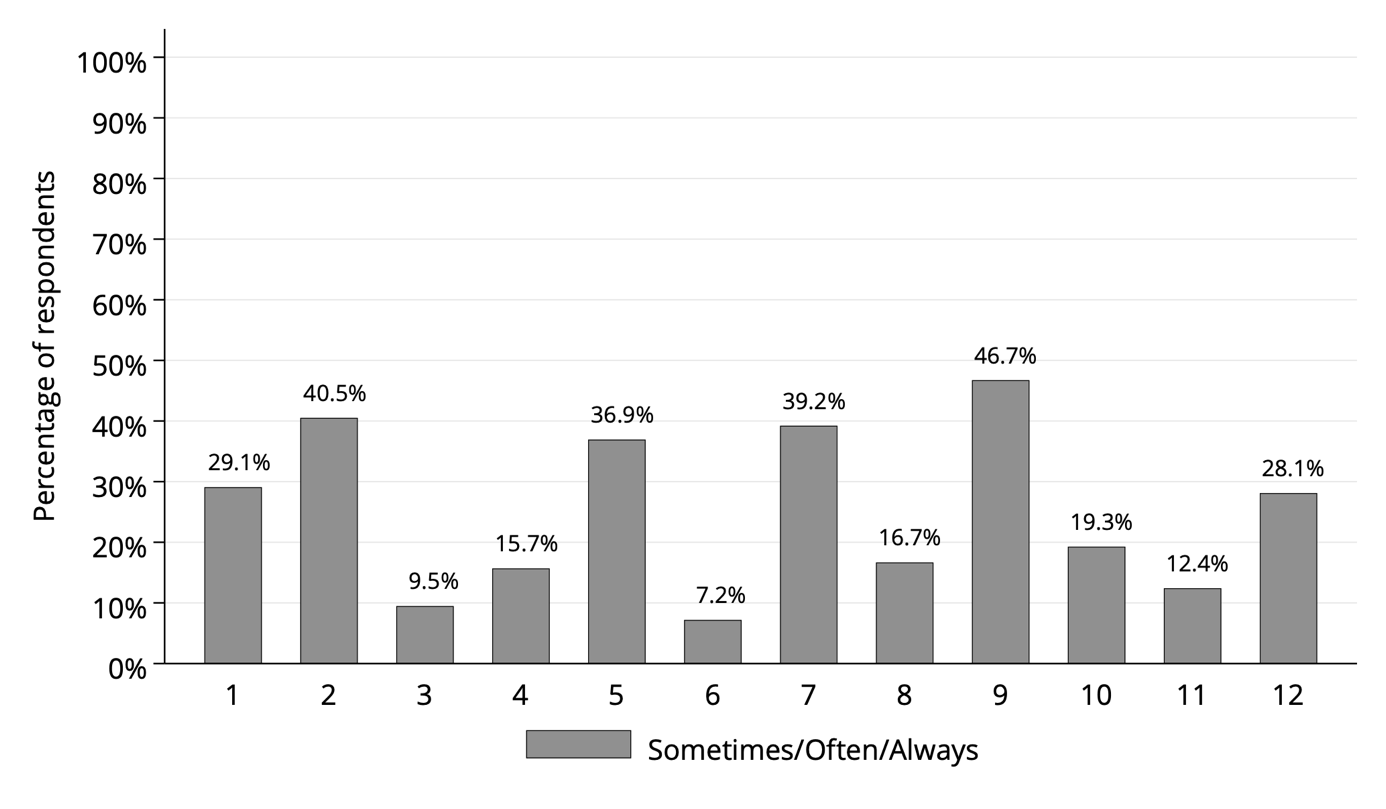


***Fig. S5. Spearman correlation matrix of GOHAI items (total sample)***

|  | Item 1 | Item 2 | Item 3 | Item 4 | Item 5 | Item 6 | Item 7 | Item 8 | Item 9 | Item 10 | Item 11 | Item 12 |
| --- | --- | --- | --- | --- | --- | --- | --- | --- | --- | --- | --- | --- |
| Item 1 | 1.00 | 0.67 | 0.23 | 0.35 | 0.58 | 0.36 | 0.20 | 0.29 | 0.38 | 0.44 | 0.40 | 0.10 |
| Item 2 |  | 1.00 | 0.18 | 0.40 | 0.63 | 0.27 | 0.28 | 0.25 | 0.35 | 0.35 | 0.40 | 0.15 |
| Item 3 |  |  | 1.00 | 0.15 | 0.18 | 0.15 | 0.14 | 0.21 | 0.17 | 0.22 | 0.23 | 0.16 |
| Item 4 |  |  |  | 1.00 | 0.28 | 0.20 | 0.15 | 0.04 | 0.13 | 0.24 | 0.20 | 0.10 |
| Item 5 |  |  |  |  | 1.00 | 0.31 | 0.27 | 0.37 | 0.41 | 0.42 | 0.44 | 0.22 |
| Item 6 |  |  |  |  |  | 1.00 | 0.29 | 0.18 | 0.26 | 0.55 | 0.52 | 0.04 |
| Item 7 |  |  |  |  |  |  | 1.00 | 0.13 | 0.33 | 0.45 | 0.28 | 0.28 |
| Item 8 |  |  |  |  |  |  |  | 1.00 | 0.19 | 0.21 | 0.12 | 0.16 |
| Item 9 |  |  |  |  |  |  |  |  | 1.00 | 0.38 | 0.23 | 0.22 |
| Item 10 |  |  |  |  |  |  |  |  |  | 1.00 | 0.56 | 0.13 |
| Item 11 |  |  |  |  |  |  |  |  |  |  | 1.00 | 0.04 |
| Item 12 |  |  |  |  |  |  |  |  |  |  |  | 1.00 |

Red: negligible inter-item correlation (rho <0.1) ; Yellow: weak inter-item correlation (rho 0.1-0.3); Green: moderate or strong inter-item correlation (rho >0.3)

***Fig S6 Path diagrams of the A) three-factor and B) one-factor Confirmatory Factor Analysis (CFA) models***

| ***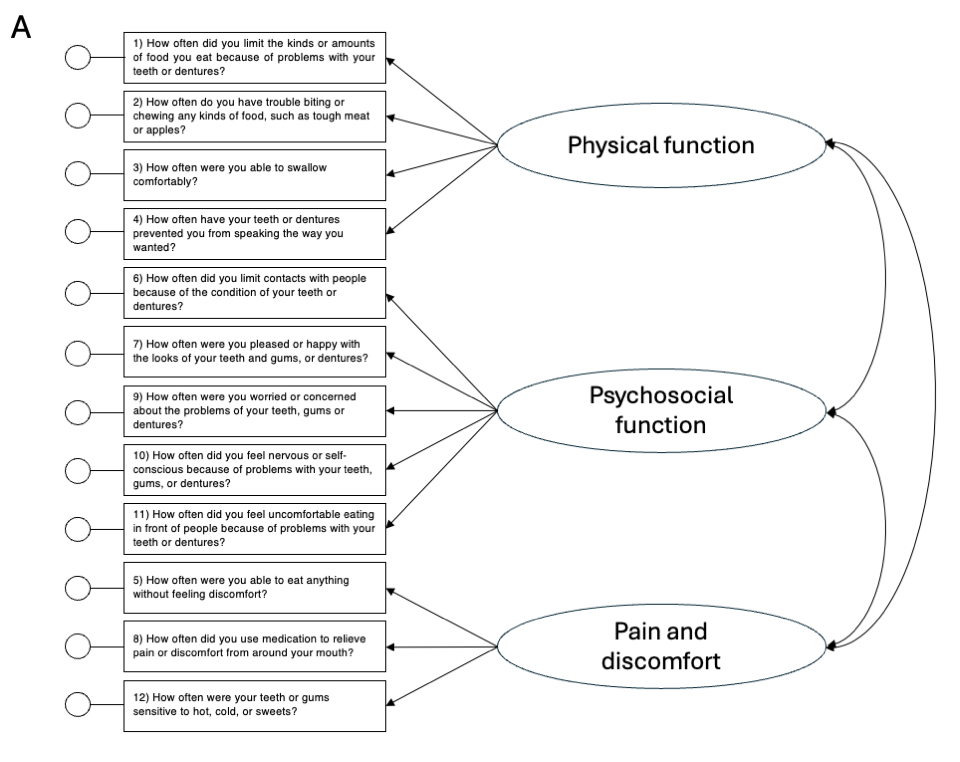*** |
| --- |
| ***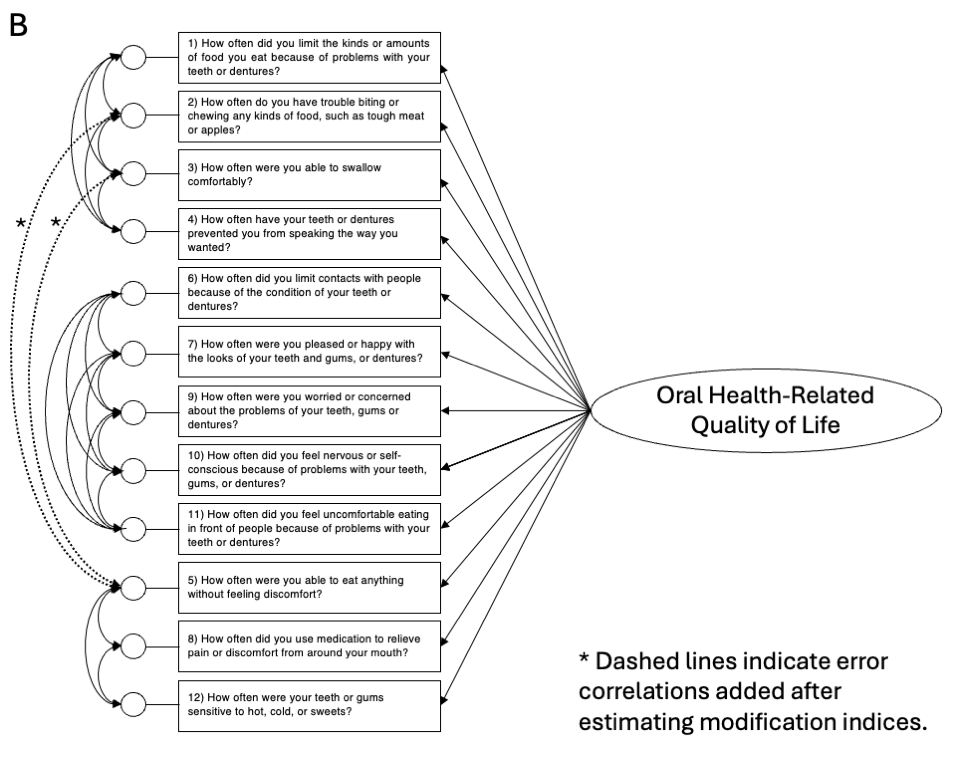*** |

Fig. S7. Comparison of factor loadings of the three- and one dimensional CFA models and Factor 1 of EFA


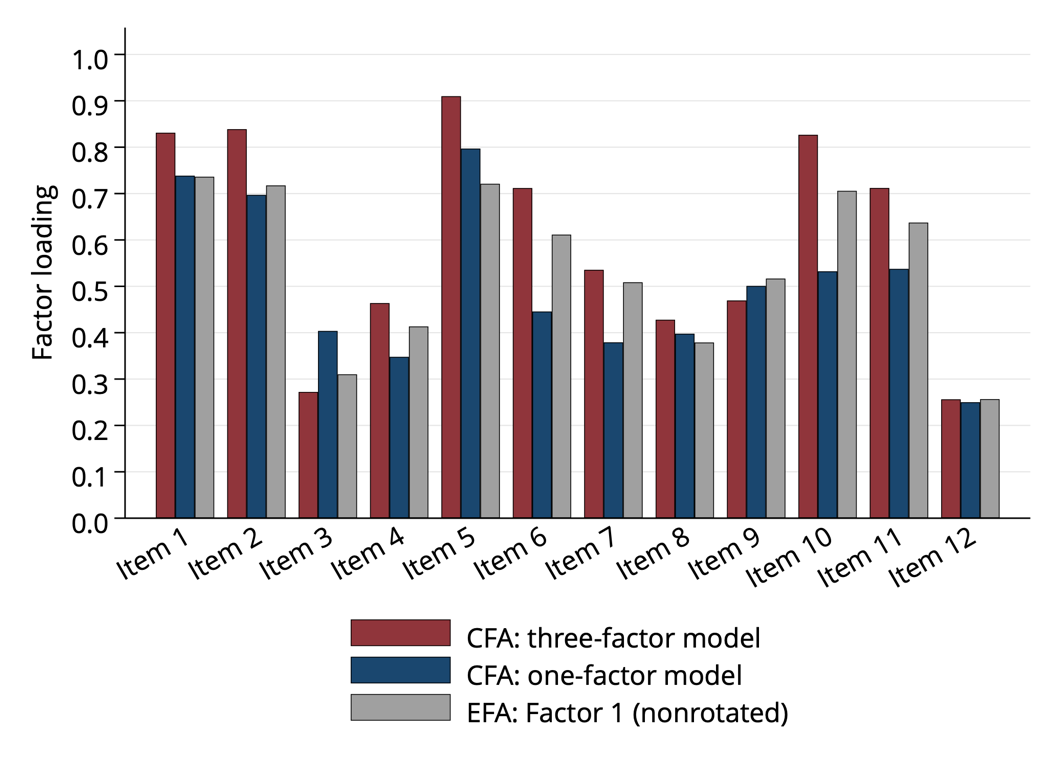


Fig. S8. CFA factor loadings, one dimensional solution


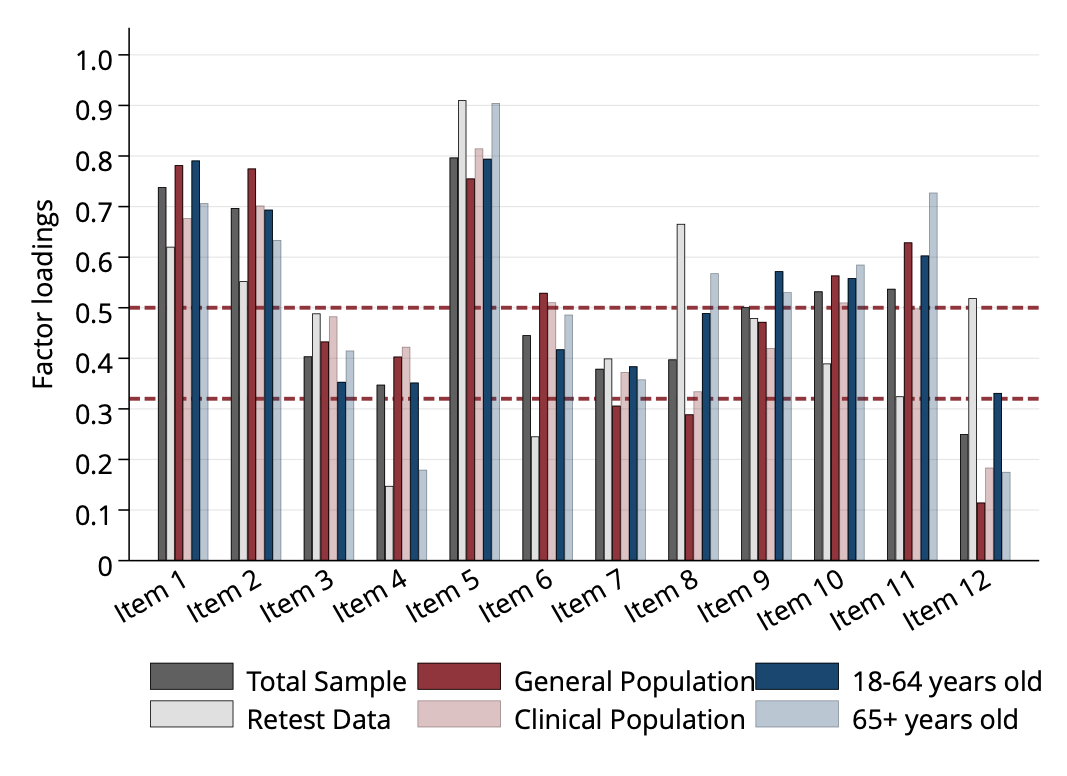


Fig. S9. Scree plot of exploratory factor analysis with BIC values overlaid (total sample)


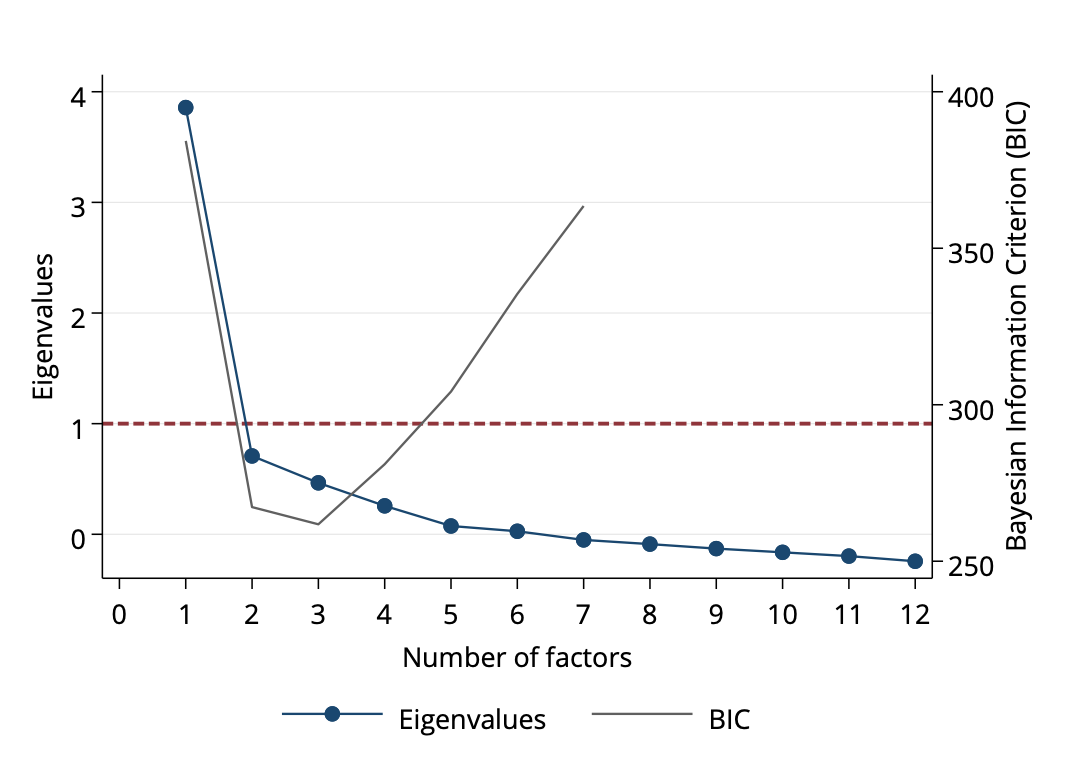


Fig. S10. Scree plot of exploratory factor analysis with BIC values overlaid (retest sample)


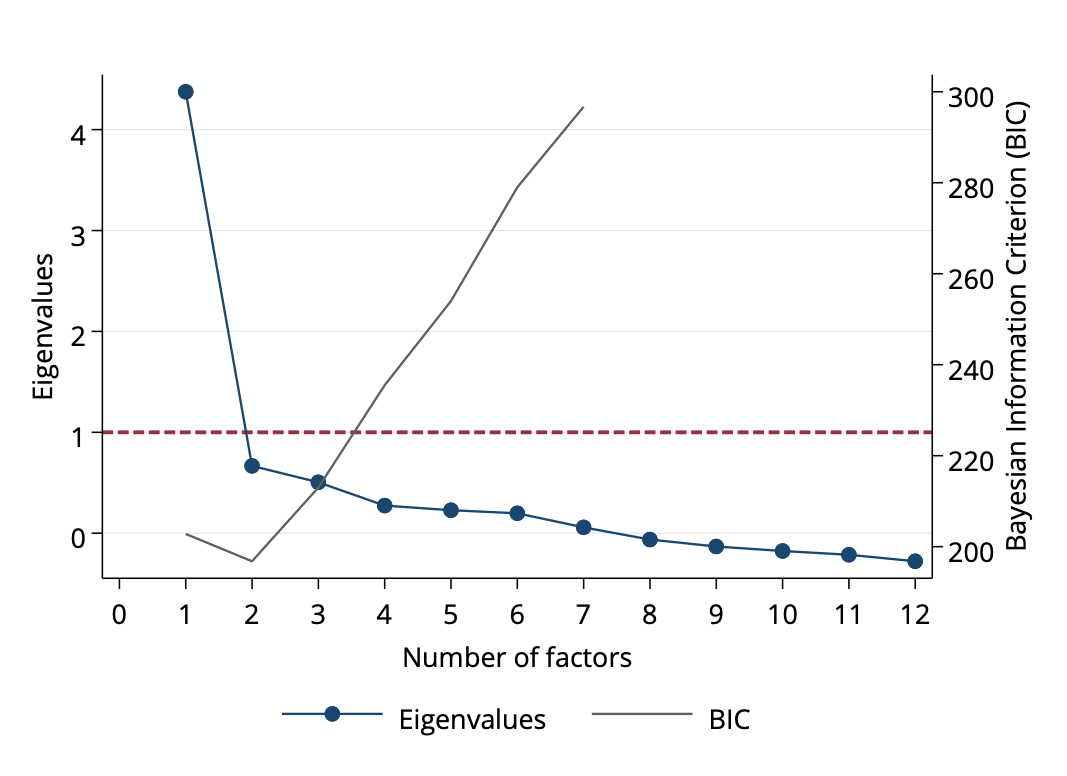


Fig S11. Exploratory factor analysis rotated solution loadings of factors 1-3 on the total sample and retest data


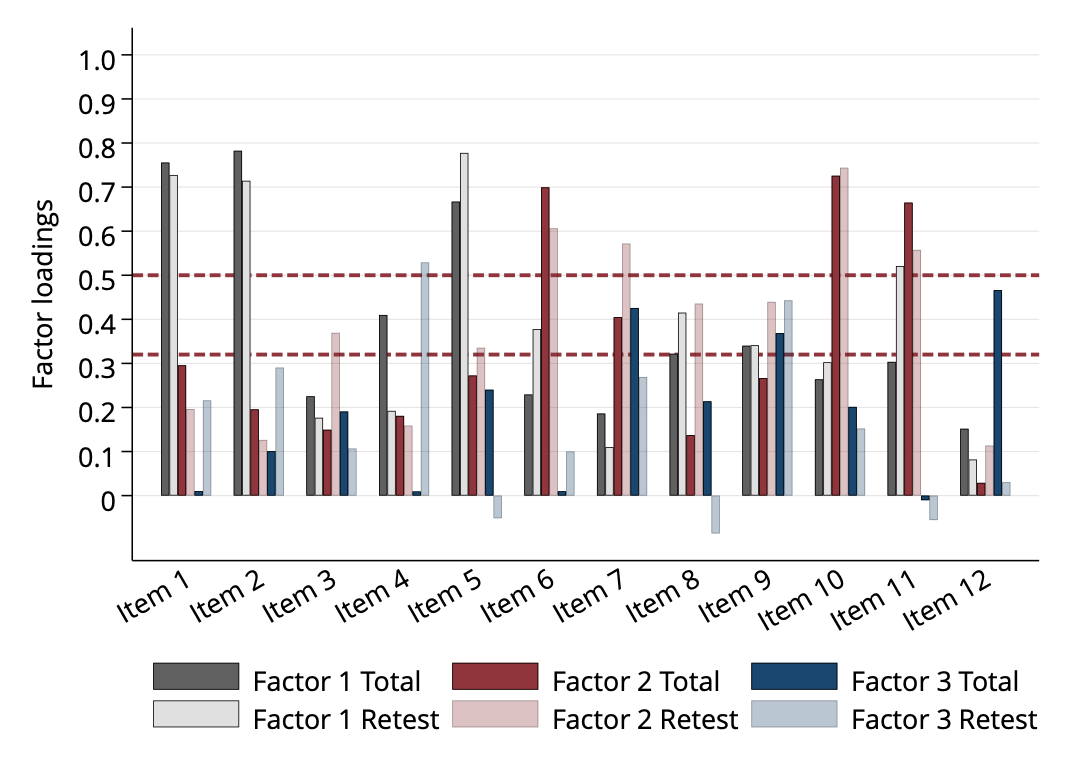


Fig S12. Exploratory factor analysis rotated solution loadings of Factor 1 across subgroups


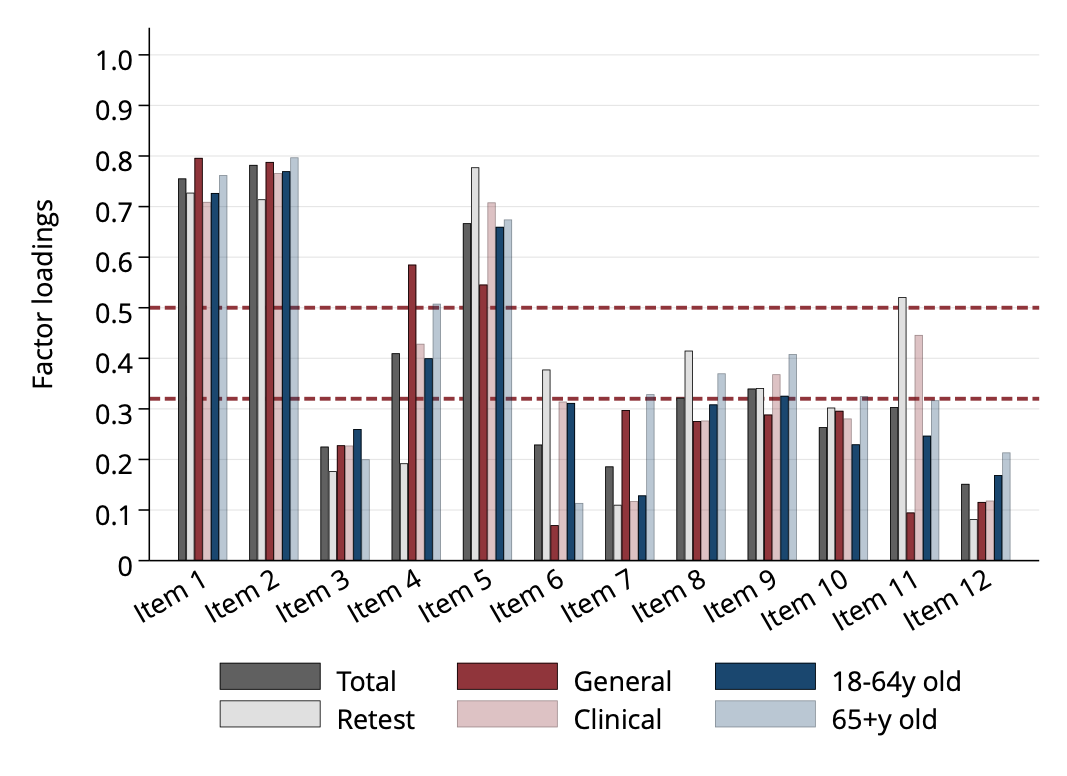


Fig S13. Exploratory factor analysis rotated solution loadings of Factor 2 across subgroups


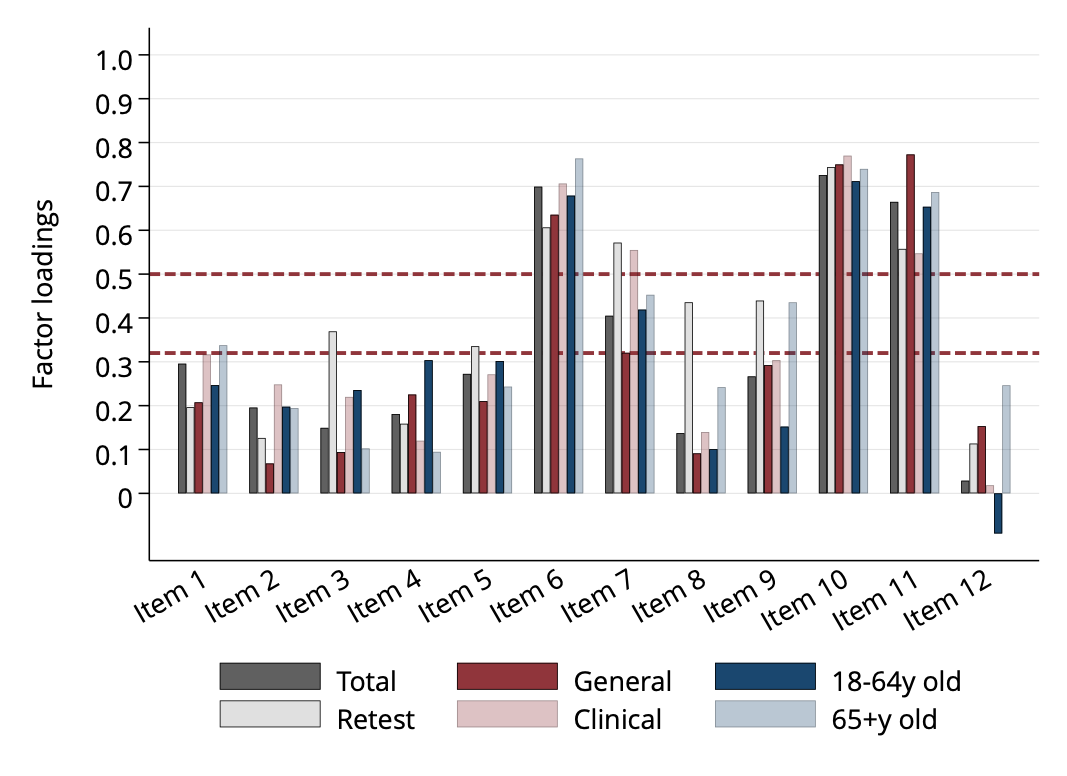


Fig S14. Exploratory factor analysis rotated solution loadings of Factor 3 across subgroups


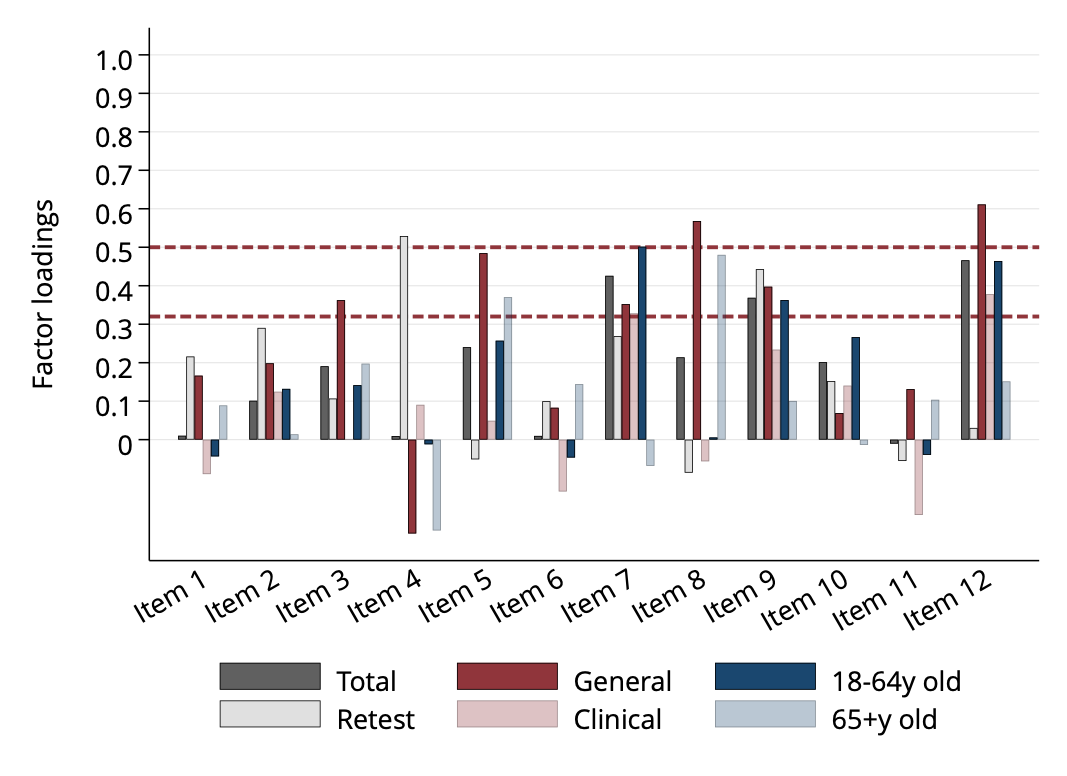

Supplement: Supplementary file 1 — Supplementary Material 1 [file 12903_2024_5198_MOESM1_ESM.docx]
